# Supplementary material for: Lipidomics reveals cell specific changes during pluripotent differentiation to neural and mesodermal lineages
Source: Mol Omics. 2025 Mar 13;21(4):259–69. doi: 10.1039/d4mo00261j (PMC11904469; doi:10.1039/d4mo00261j)
Supplement: MO-021-D4MO00261J-s001 [file MO-021-D4MO00261J-s001.pdf]

Supplementary Information (SI) for Molecular Omics.
This journal is © The Royal Society of Chemistry 2025

Contents of Report

Created by https://lipidomicstandards.org, version v2.4.0

Separation Workflow 1
Overall study design . . . 1
Lipid extraction . . . 1
Analytical platform . . . 1
Quality control . . . 1
Method qualification and validation . . . 2
Reporting . . . 2

Sample Descriptions 2
Cell Differentiation / Human / Cells . . . 2

Lipid Class Descriptions 3
1) CAR, LPC[M+H]+ / Lipid identification . . . 3
1) CAR, LPC[M+H]+ / Lipid quantification . . . 3
2) CAR, LPC[M+Na]+ / Lipid identification . . . 4
2) CAR, LPC[M+Na]+ / Lipid quantification . . . 4
3) CAR, PC[M+H]+ / Lipid identification . . . 5
3) CAR, PC[M+H]+ / Lipid quantification . . . 5
4) CAR, PC[M+Na]+ / Lipid identification . . . 6
4) CAR, PC[M+Na]+ / Lipid quantification . . . 6
5) CAR, PC P[M+H]+ / Lipid identification . . . 7
5) CAR, PC P[M+H]+ / Lipid quantification . . . 7
6) CAR, PC P[M+Na]+ / Lipid identification . . . 8
6) CAR, PC P[M+Na]+ / Lipid quantification . . . 8
7) CAR, PC[M-H]- / Lipid identification . . . 9
7) CAR, PC[M-H]- / Lipid quantification . . . 9
8) CAR, PG[M-H]- / Lipid identification . . . 10
8) CAR, PG[M-H]- / Lipid quantification . . . 10
9) CAR, PS[M-H]- / Lipid identification . . . 11
9) CAR, PS[M-H]- / Lipid quantification . . . 11
10) CAR, PE[M-H]- / Lipid identification . . . 12
10) CAR, PE[M-H]- / Lipid quantification . . . 12
11) CAR, Cer[M+CH3COO]- / Lipid identification . . . 13
11) CAR, Cer[M+CH3COO]- / Lipid quantification . . . 13
12) CAR, Cer[M+HCOO]- / Lipid identification . . . 14
12) CAR, Cer[M+HCOO]- / Lipid quantification . . . 14
13) CAR, Cer[M-H]- / Lipid identification . . . 15
13) CAR, Cer[M-H]- / Lipid quantification . . . 15
14) CAR, FA[M-H]- / Lipid identification . . . 16
14) CAR, FA[M-H]- / Lipid quantification . . . 16
15) CAR, GM3[M-H]- / Lipid identification . . . 17
15) CAR, GM3[M-H]- / Lipid quantification . . . 17
16) CAR, HexCer[M+CH3COO]- / Lipid identification . . . 18
16) CAR, HexCer[M+CH3COO]- / Lipid quantification . . . 18
17) CAR, HexCer[M+HCOO]- / Lipid identification . . . 19
17) CAR, HexCer[M+HCOO]- / Lipid quantification . . . 19
18) CAR, HexCer[M-H]- / Lipid identification . . . 20
18) CAR, HexCer[M-H]- / Lipid quantification . . . 20
19) CAR, LPC[M+CH3COO]- / Lipid identification . . . 21
19) CAR, LPC[M+CH3COO]- / Lipid quantification . . . 21
20) CAR, LPE[M-H]- / Lipid identification . . . 22
20) CAR, LPE[M-H]- / Lipid quantification . . . 22
21) CAR, LPA[M-H]- / Lipid identification . . . 23
21) CAR, LPA[M-H]- / Lipid quantification . . . 23
22) CAR, LPG[M-H]- / Lipid identification . . . 24
22) CAR, LPG[M-H]- / Lipid quantification . . . 24
23) CAR, PA[M-H]- / Lipid identification . . . 25
23) CAR, PA[M-H]- / Lipid quantification . . . 25
24) CAR, PC P[M+CH3COO]- / Lipid identification . . . 26
24) CAR, PC P[M+CH3COO]- / Lipid quantification . . . 26
25) CAR, PC O[M+CH3COO]- / Lipid identification . . . 27
25) CAR, PC O[M+CH3COO]- / Lipid quantification . . . 27
26) CAR, PI[M-H]- / Lipid identification . . . 28
26) CAR, PI[M-H]- / Lipid quantification . . . 28
27) CAR, PE P[M-H]- / Lipid identification . . . 29
27) CAR, PE P[M-H]- / Lipid quantification . . . 29
28) CAR, PE O[M-H]- / Lipid identification . . . 30
28) CAR, PE O[M-H]- / Lipid quantification . . . 30

29) CAR, SM[M-H]- / Lipid identification . . . 31
29) CAR, SM[M-H]- / Lipid quantification . . . 31
30) CAR, AC[M+H]+ / Lipid identification . . . 32
30) CAR, AC[M+H]+ / Lipid quantification . . . 32
31) CAR, ANA[M+H]+ / Lipid identification . . . 32
31) CAR, ANA[M+H]+ / Lipid quantification . . . 33
32) CAR, SE[M+NH4]+ / Lipid identification . . . 33
32) CAR, SE[M+NH4]+ / Lipid quantification . . . 33
33) CAR, Cer[M+H]+ / Lipid identification . . . 34
33) CAR, Cer[M+H]+ / Lipid quantification . . . 34
34) CAR, DG[M+NH4]+ / Lipid identification . . . 35
34) CAR, DG[M+NH4]+ / Lipid quantification . . . 35
35) CAR, LPE[M+H]+ / Lipid identification . . . 36
35) CAR, LPE[M+H]+ / Lipid quantification . . . 36
36) CAR, LPE[M+Na]+ / Lipid identification . . . 37
36) CAR, LPE[M+Na]+ / Lipid quantification . . . 37
37) CAR, PC O[M+H]+ / Lipid identification . . . 38
37) CAR, PC O[M+H]+ / Lipid quantification . . . 38
38) CAR, PC O[M+Na]+ / Lipid identification . . . 39
38) CAR, PC O[M+Na]+ / Lipid quantification . . . 39
39) CAR, PE[M+H]+ / Lipid identification . . . 40
39) CAR, PE[M+H]+ / Lipid quantification . . . 40
40) CAR, PE[M+Na]+ / Lipid identification . . . 41
40) CAR, PE[M+Na]+ / Lipid quantification . . . 41
41) CAR, SM[M+H]+ / Lipid identification . . . 42
41) CAR, SM[M+H]+ / Lipid quantification . . . 42
42) CAR, TG[M+NH4]+ / Lipid identification . . . 43
42) CAR, TG[M+NH4]+ / Lipid quantification . . . 43
43) CAR, CL[M+H]+ / Lipid identification . . . 44
43) CAR, CL[M+H]+ / Lipid quantification . . . 44

Separation Workflow

Overall study design

Title of the study	Lipidomics Reveals Cell Specific Changes During Pluripotent Differentiation to Neural and Mesodermal Lineages		
Document creation date	12/31/2024	Corresponding Email	erinmsb@unc.edu
Principal investigator	Erin Baker	Is the workflow targeted or untargeted?	Untargeted
Institution	University of North Carolina at Chapel Hill	Clinical	No

Lipid extraction

Extraction method	2-phase system	Were internal standards added prior extraction?	No
pH adjustment	None	Special conditions	-
2-phase system	Folch	Derivatization	-

Analytical platform

Ionizaton additives	Ammonium acetate	MS vendor	Agilent
Number of separation dimensions	Two dimensions	Ion source	ESI
Separation type 1	LC	MS Level	MS2
Separation mode 1 (liquid)	RP	Mass window for precursor ion isolation (in Da total isolation window)	0
Separation window for lipid analyte selection ($\pm$) in minutes	2	Mass resolution for detected ion at MS2	High resolution
Separation type 2	IMS	Resolution at m/z 200 at MS2	25000
Separation mode 2 (generic)	Drift Tube (N2)	Mass accuracy in ppm at MS2	2
Detector	Mass spectrometer	Recording mode of raw data at MS2	Centroid mode
MS type	QTOF	Was/Were additional dimension/techniques used	Yes

Quality control

Blanks	Yes	Quality control	Yes
Type of Blanks	Extraction blank, Solvent blank	Type of QC sample	Commercial sample

Method qualification and validation

Method validation	No

Reporting

Are reported raw data uploaded into repository?	Yes	Summary data	Quantification and identification data
Link to repository / ID to entry	https://doi.org/doi:10.25345/C5RW96N17	Raw data upload	Yes
Are metadata available?	Yes	Additional comments	all-ions fragmentation was performed after the IMS separation.

Sample Descriptions

Cell Differentiation / Human / Cells

Storage and collection conditions	Available	Additives	None
Provided preanalytical information	-	Were samples stored under inert gas?	No
Temperature handling original sample	4-8 °C	Additional preservation methods	No
Instant sample preparation	No	Biobank samples	No
Storage temperature	-80 °C		

Lipid Class Descriptions

1) CAR, LPC[M+H]+ / Lipid identification

Lipid class	CAR, LPC	Limit of detection	No
MS Level for identification	MS1, MS2	RT verified by standard	No
Identification level	sn Position	Separation of isobaric/isomeric interferece confirmed	No
Polarity mode	Positive	Model for separation prediction	No
Type of positive (precursor)ion	[M+H]+	Additional dimension/techniques	IMS
Fragments for identification	Fragment name: (C5H13NO,104)	CCS verified by standard	No
Isotope correction at MS1	No	How was/were the additional dimension(s) used?	For separation of isobaric/isomeric interferece at MS1 and MS2 levels
Isotope correction at MS2	No	Was a model used to predict lipid molecule separation?	No
MS1 verified by standard	No	Lipid Identification Software	Skyline
MS2 verified by standard	No	Data manipulation	-
Background check at MS1	No	Nomenclature for intact lipid molecule	Yes
Background check at MS2	No	Nomenclature for fragment ions	Yes
Did you presume assumptions for identification?	No	Further identification remarks	-
Check on:	-		

1) CAR, LPC[M+H]+ / Lipid quantification

Quantitative	No	Batch correction	No
Normalization to reference	No	Further quantification remarks	-

2) CAR, LPC[M+Na]+ / Lipid identification

Lipid class	CAR, LPC	Limit of detection	No
MS Level for identification	MS1, MS2	RT verified by standard	No
Identification level	sn Position	Separation of isobaric/isomeric interferece confirmed	No
Polarity mode	Positive	Model for separation prediction	No
Type of positive (precursor)ion	[M+Na]+	Additional dimension/techniques	IMS
Fragments for identification		CCS verified by standard	No

Fragment name
M+Na-TMA
M-HG

Isotope correction at MS1	No	How was/were the additional dimension(s) used?	For separation of isobaric/isomeric interferece in MS1 and MS2
Isotope correction at MS2	No	Was a model used to predict lipid molecule separation?	No
MS1 verified by standard	No	Lipid Identification Software	Skyline
MS2 verified by standard	No	Data manipulation	-
Background check at MS1	No	Nomenclature for intact lipid molecule	Yes
Background check at MS2	No	Nomenclature for fragment ions	N/A
Did you presume assumptions for identification?	No	Further identification remarks	-
Check on:	-		

2) CAR, LPC[M+Na]+ / Lipid quantification

Quantitative	No	Batch correction	No
Normalization to reference	No	Further quantification remarks	-

3) CAR, PC[M+H]+ / Lipid identification

Lipid class	CAR, PC	Limit of detection	No
MS Level for identification	MS1, MS2	RT verified by standard	Yes
Identification level	Molecular species level	Separation of isobaric/isomeric interferece confirmed	No
Polarity mode	Positive	Model for separation prediction	No
Type of positive (precursor)ion	[M+H]+	Additional dimension/techniques	IMS
Fragments for identification		CCS verified by standard	Yes

Fragment name
M-FA1
M-FA2

Isotope correction at MS1	No	How was/were the additional dimension(s) used?	For separation of isobaric/isomeric interferece in MS1 and MS2 dimensions
Isotope correction at MS2	No	Was a model used to predict lipid molecule separation?	No
MS1 verified by standard	No	Lipid Identification Software	Skyline
MS2 verified by standard	No	Data manipulation	-
Background check at MS1	No	Nomenclature for intact lipid molecule	No
Background check at MS2	No	Nomenclature for fragment ions	N/A
Did you presume assumptions for identification?	No	Further identification remarks	-
Check on:	-		

3) CAR, PC[M+H]+ / Lipid quantification

Quantitative	No	Batch correction	No
Normalization to reference	No	Further quantification remarks	-

4) CAR, PC[M+Na]+ / Lipid identification

Lipid class	CAR, PC	Limit of detection	No
MS Level for identification	MS1, MS2	RT verified by standard	No
Identification level	Molecular species level	Separation of isobaric/isomeric interferece confirmed	No
Polarity mode	Positive	Model for separation prediction	No
Type of positive (precursor)ion	[M+Na]+	Additional dimension/techniques	IMS
Fragments for identification		CCS verified by standard	No

Fragment name
M+Na-FA1
M+Na-FA2
M+Na-HG
M+Na-TMA
M+Na-TMA-FA1
M+Na-TMA-FA2
M-FA1
M-FA2
M-HG
M-TMA-FA1
M-TMA-FA2

Isotope correction at MS1	No	How was/were the additional dimension(s) used?	For separation of isobaric/isomeric interferece in MS1 and MS2 dimensions
Isotope correction at MS2	No	Was a model used to predict lipid molecule separation?	No
MS1 verified by standard	No	Lipid Identification Software	Skyline
MS2 verified by standard	No	Data manipulation	-
Background check at MS1	No	Nomenclature for intact lipid molecule	No
Background check at MS2	No	Nomenclature for fragment ions	N/A
Did you presume assumptions for identification?	No	Further identification remarks	-
Check on:	-		

4) CAR, PC[M+Na]+ / Lipid quantification

Quantitative	No	Batch correction	No
Normalization to reference	No	Further quantification remarks	-

5) CAR, PC P[M+H]+ / Lipid identification

Lipid class	CAR, PC P	Limit of detection	No
MS Level for identification	MS1, MS2	RT verified by standard	No
Identification level	sn Position	Separation of isobaric/isomeric interferece confirmed	No
Polarity mode	Positive	Model for separation prediction	No
Type of positive (precursor)ion	[M+H]+	Additional dimension/techniques	IMS
Fragments for identification		CCS verified by standard	No

Fragment name
M-FA1
M-pFA2

Isotope correction at MS1	No	How was/were the additional dimension(s) used?	For separation of isobaric/isomeric interferece in MS1 and MS2 dimensions
Isotope correction at MS2	No	Was a model used to predict lipid molecule separation?	No
MS1 verified by standard	No	Lipid Identification Software	Skyline
MS2 verified by standard	No	Data manipulation	-
Background check at MS1	No	Nomenclature for intact lipid molecule	No
Background check at MS2	No	Nomenclature for fragment ions	N/A
Did you presume assumptions for identification?	No	Further identification remarks	-
Check on:	-		

5) CAR, PC P[M+H]+ / Lipid quantification

Quantitative	No	Batch correction	No
Normalization to reference	No	Further quantification remarks	-

6) CAR, PC P[M+Na]+ / Lipid identification

Lipid class	CAR, PC P	Limit of detection	No
MS Level for identification	MS1, MS2	RT verified by standard	No
Identification level	sn Position	Separation of isobaric/isomeric interferece confirmed	No
Polarity mode	Positive	Model for separation prediction	No
Type of positive (precursor)ion	[M+Na]+	Additional dimension/techniques	IMS
Fragments for identification		CCS verified by standard	No

Fragment name
M+Na-HG
M+Na-TMA
M-FA1
M+Na-FA1

Isotope correction at MS1	No	How was/were the additional dimension(s) used?	For separation of isobaric/isomeric interferece in MS1 and MS2 dimensions
Isotope correction at MS2	No	Was a model used to predict lipid molecule separation?	No
MS1 verified by standard	No	Lipid Identification Software	Skyline
MS2 verified by standard	No	Data manipulation	-
Background check at MS1	No	Nomenclature for intact lipid molecule	No
Background check at MS2	No	Nomenclature for fragment ions	N/A
Did you presume assumptions for identification?	No	Further identification remarks	-
Check on:	-		

6) CAR, PC P[M+Na]+ / Lipid quantification

Quantitative	No	Batch correction	No
Normalization to reference	No	Further quantification remarks	-

7) CAR, PC[M-H]- / Lipid identification

Lipid class	CAR, PC	Limit of detection	No
MS Level for identification	MS1, MS2	RT verified by standard	No
Identification level	Molecular species level	Separation of isobaric/isomeric interferece confirmed	No
Polarity mode	Negative	Model for separation prediction	No
Type of negative (precursor)ion	[M-H]-	Additional dimension/techniques	IMS
Fragments for identification		CCS verified by standard	No

Fragment name
-(CH3+CH3COO)
-FA1 (+OH) -(CH3+CH3COO)
-FA1 (-H) -(CH3+CH3COO)
-FA2 (+OH) -(CH3+CH3COO)
-FA2 (-H) -(CH3+CH3COO)
-FA1 (+O)
-FA2 (+O)
HG(PC,224)

Isotope correction at MS1	No	How was/were the additional dimension(s) used?	For separation of isobaric/isomeric interferece in MS1 and MS2 dimensions
Isotope correction at MS2	No	Was a model used to predict lipid molecule separation?	No
MS1 verified by standard	No	Lipid Identification Software	Skyline
MS2 verified by standard	No	Data manipulation	-
Background check at MS1	No	Nomenclature for intact lipid molecule	No
Background check at MS2	No	Nomenclature for fragment ions	N/A
Did you presume assumptions for identification?	No	Further identification remarks	-
Check on:	-		

7) CAR, PC[M-H]- / Lipid quantification

Quantitative	No	Batch correction	No
Normalization to reference	No	Further quantification remarks	-

8) CAR, PG[M-H]- / Lipid identification

Lipid class	CAR, PG	Limit of detection	No
MS Level for identification	MS1, MS2	RT verified by standard	No
Identification level	Molecular species level	Separation of isobaric/isomeric interferece confirmed	No
Polarity mode	Negative	Model for separation prediction	No
Type of negative (precursor)ion	[M-H]-	Additional dimension/techniques	IMS
Fragments for identification		CCS verified by standard	No

Fragment name
-FA1 (+HO)
-FA1 (-H)
-FA1(+O)
-FA2 (+HO)
-FA2 (-H)
-FA2(+O)
GP(153)
HG(PG,171)
HG(PG,227)

Isotope correction at MS1	No	How was/were the additional dimension(s) used?	For separation of isobaric/isomeric interferece in MS1 and MS2 dimensions
Isotope correction at MS2	No	Was a model used to predict lipid molecule separation?	No
MS1 verified by standard	No	Lipid Identification Software	Skyline
MS2 verified by standard	No	Data manipulation	-
Background check at MS1	No	Nomenclature for intact lipid molecule	No
Background check at MS2	No	Nomenclature for fragment ions	N/A
Did you presume assumptions for identification?	No	Further identification remarks	-
Check on:	-		

8) CAR, PG[M-H]- / Lipid quantification

Quantitative	No	Batch correction	No
Normalization to reference	No	Further quantification remarks	-

9) CAR, PS[M-H]- / Lipid identification

Lipid class	CAR, PS	Limit of detection	No
MS Level for identification	MS1, MS2	RT verified by standard	No
Identification level	Molecular species level	Separation of isobaric/isomeric interferece confirmed	No
Polarity mode	Negative	Model for separation prediction	No
Type of negative (precursor)ion	[M-H]-	Additional dimension/techniques	IMS
Fragments for identification		CCS verified by standard	No

Fragment name
-(C3H5NO2,87)
-FA1 (+OH)
-FA1 (-H)
-FA2 (+OH)
-FA2 (-H)
-FA1 (+O)
-FA2 (+O)
GP(153)

Isotope correction at MS1	No	How was/were the additional dimension(s) used?	For separation of isobaric/isomeric interferece in MS1 and MS2 dimensions
Isotope correction at MS2	No	Was a model used to predict lipid molecule separation?	No
MS1 verified by standard	No	Lipid Identification Software	Skyline
MS2 verified by standard	No	Data manipulation	-
Background check at MS1	No	Nomenclature for intact lipid molecule	No
Background check at MS2	No	Nomenclature for fragment ions	N/A
Did you presume assumptions for identification?	No	Further identification remarks	-
Check on:	-		

9) CAR, PS[M-H]- / Lipid quantification

Quantitative	No	Batch correction	No
Normalization to reference	No	Further quantification remarks	-

10) CAR, PE[M-H]- / Lipid identification

Lipid class	CAR, PE	Limit of detection	No
MS Level for identification	MS1, MS2	RT verified by standard	No
Identification level	Molecular species level	Separation of isobaric/isomeric interferece confirmed	No
Polarity mode	Negative	Model for separation prediction	No
Type of negative (precursor)ion	[M-H]-	Additional dimension/techniques	IMS
Fragments for identification		CCS verified by standard	No

Fragment name
-FA1 (+OH)
-FA1 (+O)
-FA1 (-H)
-FA2 (+OH)
-FA2 (+O)
-FA2 (-H)
GP(153)
HG(PE,196)

Isotope correction at MS1	No	How was/were the additional dimension(s) used?	For separation of isobaric/isomeric interferece in MS1 and MS2 dimensions
Isotope correction at MS2	No	Was a model used to predict lipid molecule separation?	No
MS1 verified by standard	No	Lipid Identification Software	Skyline
MS2 verified by standard	No	Data manipulation	-
Background check at MS1	No	Nomenclature for intact lipid molecule	No
Background check at MS2	No	Nomenclature for fragment ions	N/A
Did you presume assumptions for identification?	No	Further identification remarks	-
Check on:	-		

10) CAR, PE[M-H]- / Lipid quantification

Quantitative	No	Batch correction	No
Normalization to reference	No	Further quantification remarks	-

11) CAR, Cer[M+CH3COO]- / Lipid identification

Lipid class	CAR, Cer	Limit of detection	No
MS Level for identification	MS1, MS2	RT verified by standard	No
Identification level	Species level	Separation of isobaric/isomeric interferece confirmed	No
Polarity mode	Negative	Model for separation prediction	No
Type of negative (precursor)ion	[M+CH3COO]-	Additional dimension/techniques	IMS
Fragments for identification		CCS verified by standard	No

Fragment name
FA (+C2H3N)
FA (+C2H3NO)
FA (+HN)
LCB (-C2H8NO)
LCB (-CH3O)
LCB (-H6NO)

Isotope correction at MS1	No	How was/were the additional dimension(s) used?	For separation of isobaric/isomeric interferece in MS1 and MS2 dimensions
Isotope correction at MS2	No	Was a model used to predict lipid molecule separation?	No
MS1 verified by standard	No	Lipid Identification Software	Skyline
MS2 verified by standard	No	Data manipulation	-
Background check at MS1	No	Nomenclature for intact lipid molecule	No
Background check at MS2	No	Nomenclature for fragment ions	N/A
Did you presume assumptions for identification?	No	Further identification remarks	-
Check on:	-		

11) CAR, Cer[M+CH3COO]- / Lipid quantification

Quantitative	No	Batch correction	No
Normalization to reference	No	Further quantification remarks	-

12) CAR, Cer[M+HCOO]- / Lipid identification

Lipid class	CAR, Cer	Limit of detection	No
MS Level for identification	MS1, MS2	RT verified by standard	No
Identification level	Molecular species level	Separation of isobaric/isomeric interferece confirmed	No
Polarity mode	Negative	Model for separation prediction	No
Type of negative (precursor)ion	[M+HCOO]-	Additional dimension/techniques	IMS
Fragments for identification		CCS verified by standard	No

Fragment name
FA (+C2H3N)
FA (+C2H3NO)
FA (+HN)
LCB (-C2H8NO)
LCB (-CH3O)
LCB (-H6NO)

Isotope correction at MS1	No	How was/were the additional dimension(s) used?	For separation of isobaric/isomeric interferece in MS1 and MS2 dimensions
Isotope correction at MS2	No	Was a model used to predict lipid molecule separation?	No
MS1 verified by standard	No	Lipid Identification Software	Skyline
MS2 verified by standard	No	Data manipulation	-
Background check at MS1	No	Nomenclature for intact lipid molecule	No
Background check at MS2	No	Nomenclature for fragment ions	N/A
Did you presume assumptions for identification?	No	Further identification remarks	-
Check on:	-		

12) CAR, Cer[M+HCOO]- / Lipid quantification

Quantitative	No	Batch correction	No
Normalization to reference	No	Further quantification remarks	-

13) CAR, Cer[M-H]- / Lipid identification

Lipid class	CAR, Cer	Limit of detection	No
MS Level for identification	MS1, MS2	RT verified by standard	No
Identification level	Molecular species level	Separation of isobaric/isomeric interferece confirmed	No
Polarity mode	Negative	Model for separation prediction	No
Type of negative (precursor)ion	[M-H]-	Additional dimension/techniques	IMS
Fragments for identification		CCS verified by standard	No

Fragment name
FA (+C2H3N)
FA (+C2H3NO)
FA (+HN)
LCB (-C2H8NO)
LCB (-CH3O)
LCB (-H6NO)

Isotope correction at MS1	No	How was/were the additional dimension(s) used?	For separation of isobaric/isomeric interferece in MS1 and MS2 dimensions
Isotope correction at MS2	No	Was a model used to predict lipid molecule separation?	No
MS1 verified by standard	No	Lipid Identification Software	Skyline
MS2 verified by standard	No	Data manipulation	-
Background check at MS1	No	Nomenclature for intact lipid molecule	No
Background check at MS2	No	Nomenclature for fragment ions	N/A
Did you presume assumptions for identification?	No	Further identification remarks	-
Check on:	-		

13) CAR, Cer[M-H]- / Lipid quantification

Quantitative	No	Batch correction	No
Normalization to reference	No	Further quantification remarks	-

14) CAR, FA[M-H]- / Lipid identification

Lipid class	CAR, FA	RT verified by standard	No
MS Level for identification	MS1	Separation of isobaric/isomeric interferece confirmed	No
Identification level	Species level	Model for separation prediction	No
Polarity mode	Negative	Additional dimension/techniques	IMS
Type of negative (precursor)ion	[M-H]-	CCS verified by standard	No
Isotope correction at MS1	No	How was/were the additional dimension(s) used?	For separation of isobaric/isomeric interferece in MS1 and MS2 dimensions
MS1 verified by standard	No	Was a model used to predict lipid molecule separation?	No
Background check at MS1	No	Lipid Identification Software	Skyline
Did you presume assumptions for identification?	No	Data manipulation	-
Check on:	-	Nomenclature for intact lipid molecule	No
Limit of detection	No	Further identification remarks	-

14) CAR, FA[M-H]- / Lipid quantification

Quantitative	No	Batch correction	No
Normalization to reference	No	Further quantification remarks	-

15) CAR, GM3[M-H]- / Lipid identification

Lipid class	CAR, GM3	Limit of detection	No
MS Level for identification	MS1, MS2	RT verified by standard	No
Identification level	Species level	Separation of isobaric/isomeric interferece confirmed	No
Polarity mode	Negative	Model for separation prediction	No
Type of negative (precursor)ion	[M-H]-	Additional dimension/techniques	IMS
Fragments for identification		CCS verified by standard	No

Fragment name
-HG(NHex,291)
-HG(NHex2,453)
-HG(NHex3,615)
-HG(NHex2,471)
-HG(NHex2,633)
HG(NHex, 290)

Isotope correction at MS1	No	How was/were the additional dimension(s) used?	For separation of isobaric/isomeric interferece in MS1 and Ms2 dimensions
Isotope correction at MS2	No	Was a model used to predict lipid molecule separation?	No
MS1 verified by standard	No	Lipid Identification Software	Skyline
MS2 verified by standard	No	Data manipulation	-
Background check at MS1	No	Nomenclature for intact lipid molecule	No
Background check at MS2	No	Nomenclature for fragment ions	N/A
Did you presume assumptions for identification?	No	Further identification remarks	-
Check on:	-		

15) CAR, GM3[M-H]- / Lipid quantification

Quantitative	No	Batch correction	No
Normalization to reference	No	Further quantification remarks	-

16) CAR, HexCer[M+CH3COO]- / Lipid identification

Lipid class	CAR, HexCer	Limit of detection	No
MS Level for identification	MS1, MS2	RT verified by standard	No
Identification level	Molecular species level	Separation of isobaric/isomeric interferece confirmed	No
Polarity mode	Negative	Model for separation prediction	No
Type of negative (precursor)ion	[M+CH3COO]-	Additional dimension/techniques	IMS
Fragments for identification		CCS verified by standard	No

Fragment name
-HG(Hex,162)
-HG(Hex,180)
FA (+C2H3N)
FA (+C2H3NO)
FA (+NO)
LCB (-CH3O)
LCB (-H6NO)
LCB (-C2H8NO)

Isotope correction at MS1	No	How was/were the additional dimension(s) used?	For separation of isobaric/isomeric interferece of MS1 and MS2 dimensions
Isotope correction at MS2	No	Was a model used to predict lipid molecule separation?	No
MS1 verified by standard	No	Lipid Identification Software	Skyline
MS2 verified by standard	No	Data manipulation	-
Background check at MS1	No	Nomenclature for intact lipid molecule	No
Background check at MS2	No	Nomenclature for fragment ions	N/A
Did you presume assumptions for identification?	No	Further identification remarks	-
Check on:	-		

16) CAR, HexCer[M+CH3COO]- / Lipid quantification

Quantitative	No	Batch correction	No
Normalization to reference	No	Further quantification remarks	-

17) CAR, HexCer[M+HCOO]- / Lipid identification

Lipid class	CAR, HexCer	Limit of detection	No
MS Level for identification	MS1, MS2	RT verified by standard	No
Identification level	Molecular species level	Separation of isobaric/isomeric interferece confirmed	No
Polarity mode	Negative	Model for separation prediction	No
Type of negative (precursor)ion	[M+HCOO]-	Additional dimension/techniques	IMS
Fragments for identification		CCS verified by standard	No

Fragment name
-HG(Hex,162)
-HG(Hex,180)
FA (+C2H3N)
FA (+C2H3NO)
FA (+HN)
LCB (-C2H8NO)
LCB (-CH3NO)
LCB (-H6NO)

Isotope correction at MS1	No	How was/were the additional dimension(s) used?	For separation of isobaric/isomeric interferece of MS1 and MS2 dimensions
Isotope correction at MS2	No	Was a model used to predict lipid molecule separation?	No
MS1 verified by standard	No	Lipid Identification Software	Skyline
MS2 verified by standard	No	Data manipulation	-
Background check at MS1	No	Nomenclature for intact lipid molecule	No
Background check at MS2	No	Nomenclature for fragment ions	N/A
Did you presume assumptions for identification?	No	Further identification remarks	-
Check on:	-		

17) CAR, HexCer[M+HCOO]- / Lipid quantification

Quantitative	No	Batch correction	No
Normalization to reference	No	Further quantification remarks	-

18) CAR, HexCer[M-H]- / Lipid identification

Lipid class	CAR, HexCer	Limit of detection	No
MS Level for identification	MS1, MS2	RT verified by standard	No
Identification level	Molecular species level	Separation of isobaric/isomeric interferece confirmed	No
Polarity mode	Negative	Model for separation prediction	No
Type of negative (precursor)ion	[M-H]-	Additional dimension/techniques	IMS
Fragments for identification		CCS verified by standard	No

Fragment name
FA (+C2H3O)
FA (+C2H3NO)
FA (+HN)
LCB (-C2H8NO)
LCB (-CH3O)
LCB (-H6NO)

Isotope correction at MS1	No	How was/were the additional dimension(s) used?	For separation of isobaric/isomeric interferece of MS1 and MS2 dimensions
Isotope correction at MS2	No	Was a model used to predict lipid molecule separation?	No
MS1 verified by standard	No	Lipid Identification Software	Skyline
MS2 verified by standard	No	Data manipulation	-
Background check at MS1	No	Nomenclature for intact lipid molecule	No
Background check at MS2	No	Nomenclature for fragment ions	N/A
Did you presume assumptions for identification?	No	Further identification remarks	-
Check on:	-		

18) CAR, HexCer[M-H]- / Lipid quantification

Quantitative	No	Batch correction	No
Normalization to reference	No	Further quantification remarks	-

19) CAR, LPC[M+CH3COO]- / Lipid identification

Lipid class	CAR, LPC	Limit of detection	No
MS Level for identification	MS1, MS2	RT verified by standard	No
Identification level	sn Position	Separation of isobaric/isomeric interferece confirmed	No
Polarity mode	Negative	Model for separation prediction	No
Type of negative (precursor)ion	[M+CH3COO]-	Additional dimension/techniques	IMS
Fragments for identification		CCS verified by standard	No

Fragment name
HG(PC,224)
FA1(+O)
-(CH3+CH3COO)

Isotope correction at MS1	No	How was/were the additional dimension(s) used?	For separation of isobaric/isomeric interferece of MS1 and MS2 dimensions
Isotope correction at MS2	No	Was a model used to predict lipid molecule separation?	No
MS1 verified by standard	No	Lipid Identification Software	Skyline
MS2 verified by standard	No	Data manipulation	-
Background check at MS1	No	Nomenclature for intact lipid molecule	No
Background check at MS2	No	Nomenclature for fragment ions	N/A
Did you presume assumptions for identification?	No	Further identification remarks	-
Check on:	-		

19) CAR, LPC[M+CH3COO]- / Lipid quantification

Quantitative	No	Batch correction	No
Normalization to reference	No	Further quantification remarks	-

20) CAR, LPE[M-H]- / Lipid identification

Lipid class	CAR, LPE	Limit of detection	No
MS Level for identification	MS1, MS2	RT verified by standard	No
Identification level	sn Position	Separation of isobaric/isomeric interferece confirmed	No
Polarity mode	Negative	Model for separation prediction	No
Type of negative (precursor)ion	[M-H]-	Additional dimension/techniques	IMS
Fragments for identification		CCS verified by standard	No

Fragment name
-FA1(-H)-(H2O)
-FA1(-H)
GP(153)
-FA1(+O)

Isotope correction at MS1	No	How was/were the additional dimension(s) used?	For separation of isobaric/isomeric interferece for MS1 and MS2 dimensions
Isotope correction at MS2	No	Was a model used to predict lipid molecule separation?	No
MS1 verified by standard	No	Lipid Identification Software	Skyline
MS2 verified by standard	No	Data manipulation	-
Background check at MS1	No	Nomenclature for intact lipid molecule	No
Background check at MS2	No	Nomenclature for fragment ions	N/A
Did you presume assumptions for identification?	No	Further identification remarks	-
Check on:	-		

20) CAR, LPE[M-H]- / Lipid quantification

Quantitative	No	Batch correction	No
Normalization to reference	No	Further quantification remarks	-

21) CAR, LPA[M-H]- / Lipid identification

Lipid class	CAR, LPA	Limit of detection	No
MS Level for identification	MS1, MS2	RT verified by standard	No
Identification level	Molecular species level	Separation of isobaric/isomeric interferece confirmed	No
Polarity mode	Negative	Model for separation prediction	No
Type of negative (precursor)ion	[M-H]-	Additional dimension/techniques	IMS
Fragments for identification		CCS verified by standard	No

Fragment name
GP(153)
P(79)
FA1 (+O)

Isotope correction at MS1	No	How was/were the additional dimension(s) used?	For separation of isobaric/isomeric interferece for MS1 and MS2 dimensions
Isotope correction at MS2	No	Was a model used to predict lipid molecule separation?	No
MS1 verified by standard	No	Lipid Identification Software	Skyline
MS2 verified by standard	No	Data manipulation	-
Background check at MS1	No	Nomenclature for intact lipid molecule	No
Background check at MS2	No	Nomenclature for fragment ions	N/A
Did you presume assumptions for identification?	No	Further identification remarks	-
Check on:	-		

21) CAR, LPA[M-H]- / Lipid quantification

Quantitative	No	Batch correction	No
Normalization to reference	No	Further quantification remarks	-

22) CAR, LPG[M-H]- / Lipid identification

Lipid class	CAR, LPG	Limit of detection	No
MS Level for identification	MS1, MS2	RT verified by standard	No
Identification level	Molecular species level	Separation of isobaric/isomeric interferece confirmed	No
Polarity mode	Negative	Model for separation prediction	No
Type of negative (precursor)ion	[M-H]-	Additional dimension/techniques	IMS
Fragments for identification		CCS verified by standard	No

Fragment name
GP(153)
-FA1(-H)
-FA1(+HO)
-FA1(+O)

Isotope correction at MS1	No	How was/were the additional dimension(s) used?	For separation of isobaric/isomeric interferece for MS1 and MS2 dimensions
Isotope correction at MS2	No	Was a model used to predict lipid molecule separation?	No
MS1 verified by standard	No	Lipid Identification Software	Skyline
MS2 verified by standard	No	Data manipulation	-
Background check at MS1	No	Nomenclature for intact lipid molecule	No
Background check at MS2	No	Nomenclature for fragment ions	N/A
Did you presume assumptions for identification?	No	Further identification remarks	-
Check on:	-		

22) CAR, LPG[M-H]- / Lipid quantification

Quantitative	No	Batch correction	No
Normalization to reference	No	Further quantification remarks	-

23) CAR, PA[M-H]- / Lipid identification

Lipid class	CAR, PA	Limit of detection	No
MS Level for identification	MS1, MS2	RT verified by standard	No
Identification level	Molecular species level	Separation of isobaric/isomeric interferece confirmed	No
Polarity mode	Negative	Model for separation prediction	No
Type of negative (precursor)ion	[M-H]-	Additional dimension/techniques	IMS
Fragments for identification		CCS verified by standard	No

Fragment name
GP(153)
FA1(+O)
FA1(+HO)
FA1(-H)
FA2(+O)
FA2(+HO)
FA2(-H)

Isotope correction at MS1	No	How was/were the additional dimension(s) used?	For separation of isobaric/isomeric interferece for MS1 and MS2 dimensions
Isotope correction at MS2	No	Was a model used to predict lipid molecule separation?	No
MS1 verified by standard	No	Lipid Identification Software	Skyline
MS2 verified by standard	No	Data manipulation	-
Background check at MS1	No	Nomenclature for intact lipid molecule	No
Background check at MS2	No	Nomenclature for fragment ions	N/A
Did you presume assumptions for identification?	No	Further identification remarks	-
Check on:	-		

23) CAR, PA[M-H]- / Lipid quantification

Quantitative	No	Batch correction	No
Normalization to reference	No	Further quantification remarks	-

24) CAR, PC P[M+CH3COO]- / Lipid identification

Lipid class	CAR, PC P	Limit of detection	No
MS Level for identification	MS1, MS2	RT verified by standard	No
Identification level	Molecular species level	Separation of isobaric/isomeric interferece confirmed	No
Polarity mode	Negative	Model for separation prediction	No
Type of negative (precursor)ion	[M+CH3COO]-	Additional dimension/techniques	IMS
Fragments for identification		CCS verified by standard	No

Fragment name
HG(PC)-(CH3+CH3COO)
(CH3+CH3COO)
FA1 (+OH)
FA1 (-CO)
FA1 (+O)
FA1 (-H)
FA O-[xx:x]

Isotope correction at MS1	No	How was/were the additional dimension(s) used?	For separation of isobaric/isomeric interferece for MS1 and MS2 identifications
Isotope correction at MS2	No	Was a model used to predict lipid molecule separation?	No
MS1 verified by standard	No	Lipid Identification Software	Skyline
MS2 verified by standard	No	Data manipulation	-
Background check at MS1	No	Nomenclature for intact lipid molecule	No
Background check at MS2	No	Nomenclature for fragment ions	N/A
Did you presume assumptions for identification?	No	Further identification remarks	-
Check on:	-		

24) CAR, PC P[M+CH3COO]- / Lipid quantification

Quantitative	No	Batch correction	No
Normalization to reference	No	Further quantification remarks	-

25) CAR, PC O[M+CH3COO]- / Lipid identification

Lipid class	CAR, PC O	Limit of detection	No
MS Level for identification	MS1, MS2	RT verified by standard	No
Identification level	Molecular species level	Separation of isobaric/isomeric interferece confirmed	No
Polarity mode	Negative	Model for separation prediction	No
Type of negative (precursor)ion	[M+CH3COO]-	Additional dimension/techniques	IMS
Fragments for identification		CCS verified by standard	No

Fragment name
HG(PC)-(CH3+CH3COO)
FA O-[xx:x]
FA1(+HO)
FA1(+O)
FA1-(CO)
FA1(-H)
-(CH3+CH3COO)

Isotope correction at MS1	No	How was/were the additional dimension(s) used?	For separation of isobaric/isomeric interferece for MS1 and MS2 dimensions
Isotope correction at MS2	No	Was a model used to predict lipid molecule separation?	No
MS1 verified by standard	No	Lipid Identification Software	Skyline
MS2 verified by standard	No	Data manipulation	-
Background check at MS1	No	Nomenclature for intact lipid molecule	No
Background check at MS2	No	Nomenclature for fragment ions	N/A
Did you presume assumptions for identification?	No	Further identification remarks	-
Check on:	-		

25) CAR, PC O[M+CH3COO]- / Lipid quantification

Quantitative	No	Batch correction	No
Normalization to reference	No	Further quantification remarks	-

26) CAR, PI[M-H]- / Lipid identification

Lipid class	CAR, PI	Limit of detection	No
MS Level for identification	MS1, MS2	RT verified by standard	No
Identification level	Molecular species level	Separation of isobaric/isomeric interferece confirmed	No
Polarity mode	Negative	Model for separation prediction	No
Type of negative (precursor)ion	[M-H]-	Additional dimension/techniques	IMS
Fragments for identification		CCS verified by standard	No

Fragment name
-FA1(-H)
FA1(+O)
-FA1(+HO)
-FA2(-H)
-FA2(+O)
-FA2(+HO)
GP(153)
HG(PI,241)

Isotope correction at MS1	No	How was/were the additional dimension(s) used?	For separation of isobaric/isomeric interferece in MS1 and MS2 dimensions
Isotope correction at MS2	No	Was a model used to predict lipid molecule separation?	No
MS1 verified by standard	No	Lipid Identification Software	Skyline
MS2 verified by standard	No	Data manipulation	-
Background check at MS1	No	Nomenclature for intact lipid molecule	No
Background check at MS2	No	Nomenclature for fragment ions	N/A
Did you presume assumptions for identification?	No	Further identification remarks	-
Check on:	-		

26) CAR, PI[M-H]- / Lipid quantification

Quantitative	No	Batch correction	No
Normalization to reference	No	Further quantification remarks	-

27) CAR, PE P[M-H]- / Lipid identification

Lipid class	CAR, PE P	Limit of detection	No
MS Level for identification	MS1, MS2	RT verified by standard	No
Identification level	Molecular species level	Separation of isobaric/isomeric interferece confirmed	No
Polarity mode	Negative	Model for separation prediction	No
Type of negative (precursor)ion	[M-H]-	Additional dimension/techniques	IMS
Fragments for identification		CCS verified by standard	No

Fragment name
FA2 -(CO)
-FA2(-H)
-FA2(+HO)
FA2(+O)
FA O-[xx:x]
HG(PE,196)

Isotope correction at MS1	No	How was/were the additional dimension(s) used?	For separation of isobaric/isomeric interferece in MS1 and MS21 dimensions
Isotope correction at MS2	No	Was a model used to predict lipid molecule separation?	No
MS1 verified by standard	No	Lipid Identification Software	Skyline
MS2 verified by standard	No	Data manipulation	-
Background check at MS1	No	Nomenclature for intact lipid molecule	No
Background check at MS2	No	Nomenclature for fragment ions	N/A
Did you presume assumptions for identification?	No	Further identification remarks	-
Check on:	-		

27) CAR, PE P[M-H]- / Lipid quantification

Quantitative	No	Batch correction	No
Normalization to reference	No	Further quantification remarks	-

28) CAR, PE O[M-H]- / Lipid identification

Lipid class	CAR, PE O	Limit of detection	No
MS Level for identification	MS1, MS2	RT verified by standard	No
Identification level	Molecular species level	Separation of isobaric/isomeric interferece confirmed	No
Polarity mode	Negative	Model for separation prediction	No
Type of negative (precursor)ion	[M-H]-	Additional dimension/techniques	IMS
Fragments for identification		CCS verified by standard	No

Fragment name
FA2 -(CO)
-FA2(-H)
-FA2(+HO)
FA2(+O)
GP(135)
GP(153)

Isotope correction at MS1	No	How was/were the additional dimension(s) used?	For separation of isobaric/isomeric interferece in MS1 and MS2 dimensions
Isotope correction at MS2	No	Was a model used to predict lipid molecule separation?	No
MS1 verified by standard	No	Lipid Identification Software	Skyline
MS2 verified by standard	No	Data manipulation	-
Background check at MS1	No	Nomenclature for intact lipid molecule	No
Background check at MS2	No	Nomenclature for fragment ions	N/A
Did you presume assumptions for identification?	No	Further identification remarks	-
Check on:	-		

28) CAR, PE O[M-H]- / Lipid quantification

Quantitative	No	Batch correction	No
Normalization to reference	No	Further quantification remarks	-

29) CAR, SM[M-H]- / Lipid identification

Lipid class	CAR, SM	Limit of detection	No
MS Level for identification	MS1, MS2	RT verified by standard	No
Identification level	Molecular species level	Separation of isobaric/isomeric interferece confirmed	No
Polarity mode	Negative	Model for separation prediction	No
Type of negative (precursor)ion	[M-H]-	Additional dimension/techniques	IMS
Fragments for identification		CCS verified by standard	No

Fragment name
HG(PC,168)
FA1(+O)

Isotope correction at MS1	No	How was/were the additional dimension(s) used?	For separation of isobaric/isomeric interferece in MS1 and MS2 dimensions
Isotope correction at MS2	No	Was a model used to predict lipid molecule separation?	No
MS1 verified by standard	No	Lipid Identification Software	Skyline
MS2 verified by standard	No	Data manipulation	-
Background check at MS1	No	Nomenclature for intact lipid molecule	No
Background check at MS2	No	Nomenclature for fragment ions	N/A
Did you presume assumptions for identification?	No	Further identification remarks	-
Check on:	-		

29) CAR, SM[M-H]- / Lipid quantification

Quantitative	No	Batch correction	No
Normalization to reference	No	Further quantification remarks	-

30) CAR, AC[M+H]+ / Lipid identification

Lipid class	CAR, AC	Limit of detection	No
MS Level for identification	MS1, MS2	RT verified by standard	No
Identification level	Species level	Separation of isobaric/isomeric interferece confirmed	No
Polarity mode	Positive	Model for separation prediction	No
Type of positive (precursor)ion	[M+H]+	Additional dimension/techniques	IMS
Fragments for identification	Fragment name: M-FA-TMA	CCS verified by standard	No
Isotope correction at MS1	No	How was/were the additional dimension(s) used?	For separation of isobaric/isomeric interferece in MS1 and MS2 dimensions
Isotope correction at MS2	No	Was a model used to predict lipid molecule separation?	No
MS1 verified by standard	No	Lipid Identification Software	Skyline
MS2 verified by standard	No	Data manipulation	-
Background check at MS1	No	Nomenclature for intact lipid molecule	No
Background check at MS2	No	Nomenclature for fragment ions	N/A
Did you presume assumptions for identification?	No	Further identification remarks	-
Check on:	-		

30) CAR, AC[M+H]+ / Lipid quantification

Quantitative	No	Batch correction	No
Normalization to reference	No	Further quantification remarks	-

31) CAR, ANA[M+H]+ / Lipid identification

Lipid class	CAR, ANA	RT verified by standard	No
MS Level for identification	MS1	Separation of isobaric/isomeric interferece confirmed	No
Identification level	Species level	Model for separation prediction	No
Polarity mode	Positive	Additional dimension/techniques	IMS
Type of positive (precursor)ion	[M+H]+	CCS verified by standard	No
Isotope correction at MS1	No	How was/were the additional dimension(s) used?	For separation of isobaric/isomeric interferece
MS1 verified by standard	No	Was a model used to predict lipid molecule separation?	No
Background check at MS1	No	Lipid Identification Software	Skyline
Did you presume assumptions for identification?	No	Data manipulation	-
Check on:	-	Nomenclature for intact lipid molecule	No
Limit of detection	No	Further identification remarks	-

31) CAR, ANA[M+H]+ / Lipid quantification

Quantitative	No	Batch correction	No
Normalization to reference	No	Further quantification remarks	-

32) CAR, SE[M+NH4]+ / Lipid identification

Lipid class	CAR, SE	Limit of detection	No
MS Level for identification	MS1, MS2	RT verified by standard	No
Identification level	Species level	Separation of isobaric/isomeric interferece confirmed	No
Polarity mode	Positive	Model for separation prediction	No
Type of positive (precursor)ion	[M+NH4]+	Additional dimension/techniques	IMS
Fragments for identification<br>**Fragment name**<br>-FA1(+HO)-Cholesterol(35)		CCS verified by standard	No
Isotope correction at MS1	No	How was/were the additional dimension(s) used?	For separation of isobaric/isomeric interferece in MS1 and MS2 dimensions
Isotope correction at MS2	No	Was a model used to predict lipid molecule separation?	No
MS1 verified by standard	No	Lipid Identification Software	Skyline
MS2 verified by standard	No	Data manipulation	-
Background check at MS1	No	Nomenclature for intact lipid molecule	No
Background check at MS2	No	Nomenclature for fragment ions	N/A
Did you presume assumptions for identification?	No	Further identification remarks	-
Check on:	-		

32) CAR, SE[M+NH4]+ / Lipid quantification

Quantitative	No	Batch correction	No
Normalization to reference	No	Further quantification remarks	-

33) CAR, Cer[M+H]+ / Lipid identification

Lipid class	CAR, Cer	Limit of detection	No
MS Level for identification	MS1, MS2	RT verified by standard	No
Identification level	Molecular species level	Separation of isobaric/isomeric interferece confirmed	No
Polarity mode	Positive	Model for separation prediction	No
Type of positive (precursor)ion	[M+H]+	Additional dimension/techniques	IMS
Fragments for identification		CCS verified by standard	No

Fragment name
LCB(-CH3O2)
LCB(-H3O2)
LCB(-HO)

Isotope correction at MS1	No	How was/were the additional dimension(s) used?	For separation of isobaric/isomeric interferece in MS1 and MS2 dimensions
Isotope correction at MS2	No	Was a model used to predict lipid molecule separation?	No
MS1 verified by standard	No	Lipid Identification Software	Skyline
MS2 verified by standard	No	Data manipulation	-
Background check at MS1	No	Nomenclature for intact lipid molecule	No
Background check at MS2	No	Nomenclature for fragment ions	N/A
Did you presume assumptions for identification?	No	Further identification remarks	-
Check on:	-		

33) CAR, Cer[M+H]+ / Lipid quantification

Quantitative	No	Batch correction	No
Normalization to reference	No	Further quantification remarks	-

34) CAR, DG[M+NH4]+ / Lipid identification

Lipid class	CAR, DG	Limit of detection	No
MS Level for identification	MS1, MS2	RT verified by standard	No
Identification level	Molecular species level	Separation of isobaric/isomeric interferece confirmed	No
Polarity mode	Positive	Model for separation prediction	No
Type of positive (precursor)ion	[M+NH4]+	Additional dimension/techniques	IMS
Fragments for identification		CCS verified by standard	No

Fragment name
-FA1(-H)-(H2O+NH3)
-FA2(-H)-(H2O+NH3)

Isotope correction at MS1	No	How was/were the additional dimension(s) used?	For separation of isobaric/isomeric interferece in MS1 and MS2 dimensions
Isotope correction at MS2	No	Was a model used to predict lipid molecule separation?	No
MS1 verified by standard	No	Lipid Identification Software	Skyline
MS2 verified by standard	No	Data manipulation	-
Background check at MS1	No	Nomenclature for intact lipid molecule	No
Background check at MS2	No	Nomenclature for fragment ions	N/A
Did you presume assumptions for identification?	No	Further identification remarks	-
Check on:	-		

34) CAR, DG[M+NH4]+ / Lipid quantification

Quantitative	No	Batch correction	No
Normalization to reference	No	Further quantification remarks	-

35) CAR, LPE[M+H]+ / Lipid identification

Lipid class	CAR, LPE	Limit of detection	No
MS Level for identification	MS1, MS2	RT verified by standard	No
Identification level	sn Position	Separation of isobaric/isomeric interferece confirmed	No
Polarity mode	Positive	Model for separation prediction	No
Type of positive (precursor)ion	[M+H]+	Additional dimension/techniques	IMS
Fragments for identification	Fragment name: -HG(PE,141)	CCS verified by standard	No
Isotope correction at MS1	No	How was/were the additional dimension(s) used?	For separation of isobaric/isomeric interferece of MS1 and MS2 dimensions
Isotope correction at MS2	No	Was a model used to predict lipid molecule separation?	No
MS1 verified by standard	No	Lipid Identification Software	Skyline
MS2 verified by standard	No	Data manipulation	-
Background check at MS1	No	Nomenclature for intact lipid molecule	No
Background check at MS2	No	Nomenclature for fragment ions	N/A
Did you presume assumptions for identification?	No	Further identification remarks	-
Check on:	-		

35) CAR, LPE[M+H]+ / Lipid quantification

Quantitative	No	Batch correction	No
Normalization to reference	No	Further quantification remarks	-

36) CAR, LPE[M+Na]+ / Lipid identification

Lipid class	CAR, LPE	Limit of detection	No
MS Level for identification	MS1, MS2	RT verified by standard	No
Identification level	sn Position	Separation of isobaric/isomeric interferece confirmed	No
Polarity mode	Positive	Model for separation prediction	No
Type of positive (precursor)ion	[M+Na]+	Additional dimension/techniques	IMS
Fragments for identification		CCS verified by standard	No
Isotope correction at MS1	No	How was/were the additional dimension(s) used?	For separation of isobaric/isomeric interferece in MS1 and MS2 dimensions
Isotope correction at MS2	No	Was a model used to predict lipid molecule separation?	No
MS1 verified by standard	No	Lipid Identification Software	Skyline
MS2 verified by standard	No	Data manipulation	-
Background check at MS1	No	Nomenclature for intact lipid molecule	No
Background check at MS2	No	Nomenclature for fragment ions	N/A
Did you presume assumptions for identification?	No	Further identification remarks	-
Check on:	-		

Fragment name
M-HG
M+ Na-az

36) CAR, LPE[M+Na]+ / Lipid quantification

Quantitative	No	Batch correction	No
Normalization to reference	No	Further quantification remarks	-

37) CAR, PC O[M+H]+ / Lipid identification

Lipid class	CAR, PC O	Limit of detection	No
MS Level for identification	MS1, MS2	RT verified by standard	No
Identification level	Molecular species level	Separation of isobaric/isomeric interferece confirmed	No
Polarity mode	Positive	Model for separation prediction	No
Type of positive (precursor)ion	[M+H]+	Additional dimension/techniques	IMS
Fragments for identification		CCS verified by standard	No

Fragment name
M-FA1
M-oFA2

Isotope correction at MS1	No	How was/were the additional dimension(s) used?	For separation of isobaric/isomeric interferece in MS1 and MS2 dimensions
Isotope correction at MS2	No	Was a model used to predict lipid molecule separation?	No
MS1 verified by standard	No	Lipid Identification Software	Skyline
MS2 verified by standard	No	Data manipulation	-
Background check at MS1	No	Nomenclature for intact lipid molecule	No
Background check at MS2	No	Nomenclature for fragment ions	N/A
Did you presume assumptions for identification?	No	Further identification remarks	-
Check on:	-		

37) CAR, PC O[M+H]+ / Lipid quantification

Quantitative	No	Batch correction	No
Normalization to reference	No	Further quantification remarks	-

38) CAR, PC O[M+Na]+ / Lipid identification

Lipid class	CAR, PC O	Limit of detection	No
MS Level for identification	MS1, MS2	RT verified by standard	No
Identification level	Molecular species level	Separation of isobaric/isomeric interferece confirmed	No
Polarity mode	Positive	Model for separation prediction	No
Type of positive (precursor)ion	[M+Na]+	Additional dimension/techniques	IMS
Fragments for identification		CCS verified by standard	No

Fragment name
M+Na-HG
M+Na-TMA
M-FA

Isotope correction at MS1	No	How was/were the additional dimension(s) used?	For separation of isobaric/isomeric interferece in MS1 and MS2 dimensions
Isotope correction at MS2	No	Was a model used to predict lipid molecule separation?	No
MS1 verified by standard	No	Lipid Identification Software	Skyline
MS2 verified by standard	No	Data manipulation	-
Background check at MS1	No	Nomenclature for intact lipid molecule	No
Background check at MS2	No	Nomenclature for fragment ions	N/A
Did you presume assumptions for identification?	No	Further identification remarks	-
Check on:	-		

38) CAR, PC O[M+Na]+ / Lipid quantification

Quantitative	No	Batch correction	No
Normalization to reference	No	Further quantification remarks	-

39) CAR, PE[M+H]+ / Lipid identification

Lipid class	CAR, PE	Limit of detection	No
MS Level for identification	MS1, MS2	RT verified by standard	No
Identification level	Molecular species level	Separation of isobaric/isomeric interferece confirmed	No
Polarity mode	Positive	Model for separation prediction	No
Type of positive (precursor)ion	[M+H]+	Additional dimension/techniques	IMS
Fragments for identification		CCS verified by standard	No

Fragment name
-HG(PE,141)
FA1 (+O)
FA2 (+O)

Isotope correction at MS1	No	How was/were the additional dimension(s) used?	For separation of isobaric/isomeric interferece in MS1 and MS2 dimensions
Isotope correction at MS2	No	Was a model used to predict lipid molecule separation?	No
MS1 verified by standard	No	Lipid Identification Software	Skyline
MS2 verified by standard	No	Data manipulation	-
Background check at MS1	No	Nomenclature for intact lipid molecule	No
Background check at MS2	No	Nomenclature for fragment ions	N/A
Did you presume assumptions for identification?	No	Further identification remarks	-
Check on:	-		

39) CAR, PE[M+H]+ / Lipid quantification

Quantitative	No	Batch correction	No
Normalization to reference	No	Further quantification remarks	-

40) CAR, PE[M+Na]+ / Lipid identification

Lipid class	CAR, PE	Limit of detection	No
MS Level for identification	MS1, MS2	RT verified by standard	No
Identification level	Molecular species level	Separation of isobaric/isomeric interferece confirmed	No
Polarity mode	Positive	Model for separation prediction	No
Type of positive (precursor)ion	[M+Na]+	Additional dimension/techniques	IMS
Fragments for identification		CCS verified by standard	No

Fragment name
M-HG
M+Na-HG
M+Na-az
M+Na-C2H5N-FA1
M+Na-C2H5N-FA2

Isotope correction at MS1	No	How was/were the additional dimension(s) used?	For separation of isobaric/isomeric interference in MS1 and MS2 dimensions
Isotope correction at MS2	No	Was a model used to predict lipid molecule separation?	No
MS1 verified by standard	No	Lipid Identification Software	Skyline
MS2 verified by standard	No	Data manipulation	-
Background check at MS1	No	Nomenclature for intact lipid molecule	No
Background check at MS2	No	Nomenclature for fragment ions	N/A
Did you presume assumptions for identification?	No	Further identification remarks	-
Check on:	-		

40) CAR, PE[M+Na]+ / Lipid quantification

Quantitative	No	Batch correction	No
Normalization to reference	No	Further quantification remarks	-

41) CAR, SM[M+H]+ / Lipid identification

Lipid class	CAR, SM	Limit of detection	No
MS Level for identification	MS1, MS2	RT verified by standard	No
Identification level	Molecular species level	Separation of isobaric/isomeric interferece confirmed	No
Polarity mode	Positive	Model for separation prediction	No
Type of positive (precursor)ion	[M+H]+	Additional dimension/techniques	IMS
Fragments for identification	Fragment name: LCB(-H3O2)	CCS verified by standard	No
Isotope correction at MS1	No	How was/were the additional dimension(s) used?	For separation of isobaric/isomeric interferece in MS1 and MS2 dimensions
Isotope correction at MS2	No	Was a model used to predict lipid molecule separation?	No
MS1 verified by standard	No	Lipid Identification Software	Skyline
MS2 verified by standard	No	Data manipulation	-
Background check at MS1	No	Nomenclature for intact lipid molecule	No
Background check at MS2	No	Nomenclature for fragment ions	N/A
Did you presume assumptions for identification?	No	Further identification remarks	-
Check on:	-		

41) CAR, SM[M+H]+ / Lipid quantification

Quantitative	No	Batch correction	No
Normalization to reference	No	Further quantification remarks	-

42) CAR, TG[M+NH4]+ / Lipid identification

Lipid class	CAR, TG	Limit of detection	No
MS Level for identification	MS1, MS2	RT verified by standard	No
Identification level	Molecular species level	Separation of isobaric/isomeric interferece confirmed	No
Polarity mode	Positive	Model for separation prediction	No
Type of positive (precursor)ion	[M+NH4]+	Additional dimension/techniques	IMS
Fragments for identification		CCS verified by standard	No

Fragment name
-FA3(+HO)-(NH3)
-FA2(+HO)-(NH3)
-FA1(+HO)-(NH3)
FA3
FA2
FA1

Isotope correction at MS1	No	How was/were the additional dimension(s) used?	For separation of isobaric/isomeric interferece of MS1 and MS2 dimensions
Isotope correction at MS2	No	Was a model used to predict lipid molecule separation?	No
MS1 verified by standard	No	Lipid Identification Software	Skyline
MS2 verified by standard	No	Data manipulation	-
Background check at MS1	No	Nomenclature for intact lipid molecule	No
Background check at MS2	No	Nomenclature for fragment ions	N/A
Did you presume assumptions for identification?	No	Further identification remarks	-
Check on:	-		

42) CAR, TG[M+NH4]+ / Lipid quantification

Quantitative	No	Batch correction	No
Normalization to reference	No	Further quantification remarks	-

43) CAR, CL[M+H]+ / Lipid identification

Lipid class	CAR, CL	RT verified by standard	No
MS Level for identification	MS1	Separation of isobaric/isomeric interferece confirmed	No
Identification level	Species level	Model for separation prediction	No
Polarity mode	Positive	Additional dimension/techniques	IMS
Type of positive (precursor)ion	[M+H]+	CCS verified by standard	No
Isotope correction at MS1	No	How was/were the additional dimension(s) used?	For separation of isobaric/isomeric interferece
MS1 verified by standard	No	Was a model used to predict lipid molecule separation?	No
Background check at MS1	No	Lipid Identification Software	Skyline
Did you presume assumptions for identification?	No	Data manipulation	-
Check on:	-	Nomenclature for intact lipid molecule	No
Limit of detection	No	Further identification remarks	-

43) CAR, CL[M+H]+ / Lipid quantification

Quantitative	No	Batch correction	No
Normalization to reference	No	Further quantification remarks	-
